# SNPs in bone-related miRNAs are associated with the osteoporotic phenotype

**DOI:** 10.1038/s41598-017-00641-7

**Published:** 2017-03-31

**Authors:** Laura De-Ugarte, Enrique Caro-Molina, Maria Rodríguez-Sanz, Miguel Angel García-Pérez, José M. Olmos, Manuel Sosa-Henríquez, Ramón Pérez-Cano, Carlos Gómez-Alonso, Luis Del Rio, Jesús Mateo-Agudo, José Antonio Blázquez-Cabrera, Jesús González-Macías, Javier del Pino-Montes, Manuel Muñoz-Torres, Manuel Diaz-Curiel, Jorge Malouf, Antonio Cano, José Luis Pérez-Castrillon, Xavier Nogues, Natalia Garcia-Giralt, Adolfo Diez-Perez

**Affiliations:** 1 IMIM (Hospital del Mar Medical Research Institute), Universitat Autònoma de Barcelona, CIBERFES, RETICEF (ISCIII), Barcelona, Spain; 2 Department of Genetics and Institute of Health Research INCLIVA, University of Valencia, Valencia, Spain; 3 Department of Internal Medicine, Hospital Universitario Marqués de Valdecilla-IDIVAL/Hospital de Torrelavega, Universidad de Cantabria. RETICEF, Santander, Spain; 4 Unidad Metabólica Ósea, Hospital Universitario Insular, Universidad de Las Palmas de Gran Canaria, Canarias, Spain; 5 Departamento de Medicina (USE), UGC Medicina Interna, Hospital Universitario Virgen Macarena, Seville, Spain; 6 Servicio de Metabolismo Óseo y Mineral, Hospital Universitario Central de Asturias, Oviedo, Spain; 7 CETIR Grup Mèdic, RETICEF, Barcelona, Spain; 8 Servicio COT, Hospital Universitario Miguel Servet, Zaragoza, Spain; 9 Servicio de Medicina Interna, Complejo Hospitalario Universitario de Albacete, Albacete, Spain; 10 Departamento de Medicina Interna, H. Marqués de Valdecilla, Universidad de Cantabria, IDIVAL, RETICEF, Santander, Spain; 11 Servicio de Reumatología. Hospital Universitario de Salamanca, RETICEF (ISCIII), IBSAL (Biomedical Research Institute of Salamanca), Salamanca, Spain; 12 UGC Endocrinologia y Nutrición. Hospital Universitario San Cecilio. Granada, RETICEF, Ibs, Granada, Spain; 13 Unidad de Enfermedades Metabólicas Óseas. Servicio de Medicina Interna. Fundacion Jimenez Diaz, Madrid, Spain; 14 Hospital de la Santa Creu I Sant Pau. Institut d’Investigació Biomèdica Sant Pau, Barcelona, Spain; 15 Department of Pediatrics, Obstetrics and Gynecology, University of Valencia, INCLIVA, Valencia, Spain; 16 Hospital Universitario Río Hortega, Valladolid, Spain

## Abstract

Biogenesis and function of microRNAs can be influenced by genetic variants in the pri-miRNA sequences leading to phenotypic variability. This study aims to identify single nucleotide polymorphisms (SNPs) affecting the expression levels of bone-related mature microRNAs and thus, triggering an osteoporotic phenotype. An association analysis of SNPs located in pri-miRNA sequences with bone mineral density (BMD) was performed in the OSTEOMED2 cohort (n = 2183). Functional studies were performed for assessing the role of BMD-associated miRNAs in bone cells. Two SNPs, rs6430498 in the miR-3679 and rs12512664 in the miR-4274, were significantly associated with femoral neck BMD. Further, we measured these BMD-associated microRNAs in trabecular bone from osteoporotic hip fractures comparing to non-osteoporotic bone by qPCR. Both microRNAs were found overexpressed in fractured bone. Increased matrix mineralization was observed after miR-3679-3p inhibition in human osteoblastic cells. Finally, genotypes of rs6430498 and rs12512664 were correlated with expression levels of miR-3679 and miR-4274, respectively, in osteoblasts. In both cases, the allele that generated higher microRNA expression levels was associated with lower BMD values. In conclusion, two osteoblast-expressed microRNAs, miR-3679 and miR-4274, were associated with BMD; their overexpression could contribute to the osteoporotic phenotype. These findings open new areas for the study of bone disorders.

## Introduction

MicroRNAs (miRNAs) have opened a new field of research for complex diseases with a genetic basis. These small non-coding RNAs inhibit the expression of target mRNAs by binding to their 3′-untranslated regions (3′UTRs). These molecules have added a new step of complexity in gene regulation, but may also help to increase our understanding of many multifactorial diseases that have been a mystery up to now.

miRNAs have been extensively studied in bone research, particularly their relationship to osteoporosis^1–3^. These studies clearly showed altered miRNAs profiling in serum from patients with osteoporosis, as well as in bone tissue after osteoporotic (OP) fracture. However, these miRNA expression signatures observed in patients with osteoporosis do not provide evidence of causality because the altered pattern could be a consequence of the disease or even unrelated to the pathogenesis.

Another approach in miRNAs studies is the association analysis between one SNP within a candidate miRNA (miR-SNP) or in a miRNA target site and one disease related-outcome. In this case, the associated variant is likely involved in the pathophysiology or confers susceptibility to develop the disease. Moreover, many evidences suggest that the genetics of complex traits are attributable to genetic variations that modulate gene expression, rather than the variations resulting in protein structure changes^4^. However, functional assays are needed in order to elucidate the role of the associated variants in the pathophysiology of the disease since the SNP could be in linkage disequilibrium with the true functional variant.

The aim of this study was to identify SNPs within candidate miRNAs in order to perform an association study between those SNPs and bone mineral density (BMD), the main outcome used to define osteoporosis. First, we searched for miR-SNPs in the primary miRNA transcript (pri-miRNA), which has a hairpin structure with a terminal loop and two single-stranded flanking regions. The pri-miRNAs are recognized and cleaved by the Drosha and DCGR8 complex, resulting in a shorter structure called pre-miRNA^5^. In this step, the sequences at the unpaired flanking arms and within the hairpin double-stranded stem structure are crucial to correct binding and cleavage by the Drosha-DCGR8 complex. Thus, the existence of genetic variants within the pri-miRNA sequences could lead to an alteration of the hairpin structure, affecting molecular processing and the underlying miRNA maturation^6^. Changes in miRNA maturation would trigger changes in miRNA abundancy, and consequently a deregulation of the expression levels of target genes. Supporting this idea, large-scale *in silico* analyses of SNPs in human miRNA genes have demonstrated lower SNP densities in the miRNA sequence than their flanking regions or the human genome^7^. Hence, our study was based on the detection and subsequent genetic association analysis of putative functional miR-SNPs. Furthermore, associated miR-SNPs were explored in bone cells in order to validate the association with the OP phenotype.

## Results

**Figure 1:**
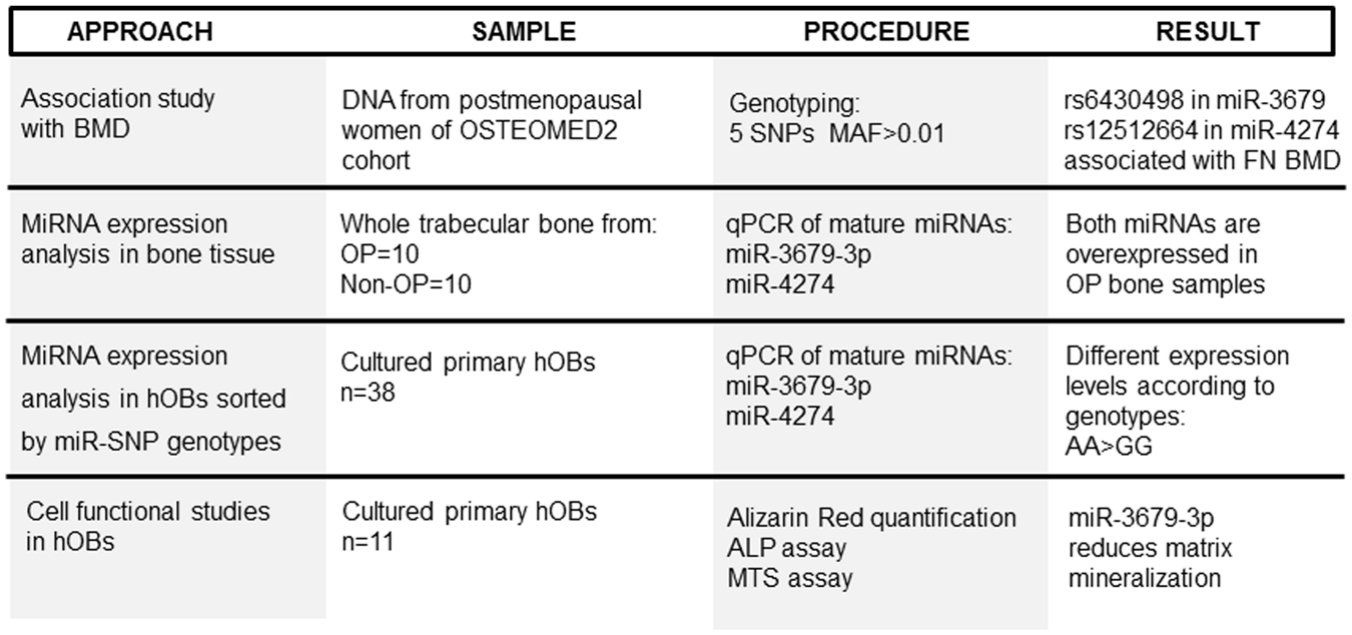
Schematic overview of the whole procedures, samples and results of the study.

An overall overview of methodology and results is schematized in Fig. 1.

### Association analysis with BMD

The first approach used in our study was to identify functional variants within microRNAs involved in bone metabolism. The minor allele frequency (MAF) of many of the miR-SNPs found in databases has not been assessed in European population; therefore, a validation step was necessary. In this regard, SNPs in microRNA sequences were firstly validated as polymorphic in patients from BARCOS subcohort^8^. Of the 53 miR-SNPs tested, 14 were validated in this cohort. However, only 5 SNPs had a MAF > 0.01 and were finally genotyped in the OSTEOMED2 cohort for their association analysis with LS (lumbar spine) BMD and FN (femoral neck) BMD (Table 1).

**Table 1:** Validation of miR-SNPs for the BMD association analysis.

TARGET GENE	miRNA	SNP	MAFB	Association with BMD
*ESR1*	miR-106b	rs72631827	Monomorphic	
	miR-130b	rs72631822	Monomorphic	
	miR-148b	rs74878365	Monomorphic	
	miR-18a	rs41275866	Monomorphic	
	miR-222	rs72631825	Monomorphic	
	miR-373	rs80338016	Monomorphic	
	miR-520c	rs7255628	Monomorphic	
	miR-93	rs72631824	Monomorphic	
	miR-96	rs41274239	0.0033	MAF < 0.01
		rs73159662	0.0058	
*TGFB2*	miR-141	rs34385807	Monomorphic	
	miR-149	rs71428439	Monomorphic	
	miR-182	rs77586312	Monomorphic	
		rs75953509	Monomorphic	
		rs80041074	0.0033	MAF < 0.01
	miR-199b	rs72631835	Monomorphic	
	miR-193a	rs60406007	Monomorphic	
	miR-200b	rs72563729	Monomorphic	
	miR-33a	rs77809319	Monomorphic	
	miR-431	rs76090066	0.00083	MAF < 0.01
		rs128840′05	Monomorphic	
	miR-590	rs6971711	Monomorphic	
	miR-7-1	rs76662330	Monomorphic	
	miR-7-2	rs41276930	0.005	MAF < 0.01
		rs75737367	Monomorphic	
*PTH1R*	miR-339	rs13232101	Monomorphic	
		rs72631820	Monomorphic	
		rs72631831	Monomorphic	
*RUNX2*	miR-122	rs41292412	0.0033	MAF < 0.01
	miR-154	rs41286570	0.0004	
*IL6R*	miR-124-2	rs72631829	Monomorphic	
	miR-124-3	rs34059726	Monomorphic	
	miR-125a	rs12975333	Monomorphic	
	miR-140	rs7205289	Monomorphic	
	miR-320d-1	rs74826059	Monomorphic	
	**miR-499**	**rs3746444**	**0.21**	**Not associated**
		rs7267163	0.0025	MAF < 0.01
*LRP5*	**miR-27a**	**rs11671784**	**0.0162**	**Not associated**
*IL6*	**miR-146a**	**rs2910164**	**0.26**	**Not associated**
	miR-146b	rs76149940	Monomorphic	
	miR-202	rs12355840	Monomorphic	
	miR-365-2	rs35143473	Monomorphic	
*VDR*	miR-10a	rs72631828	Monomorphic	
	miR-223	rs34952329	Monomorphic	
*CYP24A1*	miR-30b	rs111424617	Monomorphic	
	miR-30e	rs112439044	Monomorphic	
	miR-183	rs72631833	Monomorphic	
		rs41281222	Monomorphic	
	miR-101-2	rs78851134	0.0004	MAF < 0.01
Highly expressed in HObs	miR-1282	rs11269	Monomorphic	
	**miR-3679**	**rs6430498**	**0.35**	**Associated with FN BMD**
		rs10175383	Monomorphic	
	**miR-4274**	rs12512664	**0.47**	**Associated with FN BMD**

MAFB; Minor allele frequency in BARCOS cohort; SNPs with MAF<0.01 were excluded for genotyping. In bold; Validated SNPs for genotyping in total OSTEOMED2 cohort.

SNP rs6430498 in the miR-3679 was significantly associated with FN BMD in a log-additive (*p* value = 0.036, Beta coefficient [95% CI] = −0.008 [−0.015 to −0.001] and recessive model (*p* value = 0.021, Beta coefficient [95% CI] = −0.017 [−0.032 to −0.003]); and the SNP rs12512664 in the miR-4274 was also significantly associated with FN BMD in a log-additive (*p* value = 0.016, Beta coefficient [95% CI] = 0.008 [0.002 to 0.015] and recessive model (*p* value = 0.01, Beta coefficient [95% CI] = 0.015 [0.004 to 0.027). For both SNPs, the genotyping efficiency was approximately 98%. The A alleles for rs6430498 (minority allele) and rs12512664 (majority allele) were found to be associated with lower BMD values.

### Quantification of miRNA expression levels in total FN bone samples

The anthropometric features of the OP and Control groups were shown in Table 2. Using the Mann–Whitney U test, no statistical differences in any features were observed between both groups.

**Table 2:** Patient characteristics for osteoporotic fracture and non-osteoporotic groups.

Bicological groups	n	Age (Mean ± SD)	BMI (kg/m^2^) (Mean ± SD)	BMD (g/cm^2^) (Mean ± SD)
Osteoporotic	10	75.6 ± 6.38	27.11 ± 2.94	Fragility fracture
Non-osteoporotic	10	71.7 ± 7.36	27.42 ± 3.15	0.882 ± 0.137

Abbreviations: SD: Standard Deviation; BMI: Body Mass Index; BMD: Bone Mineral Density.

**Figure 2:**
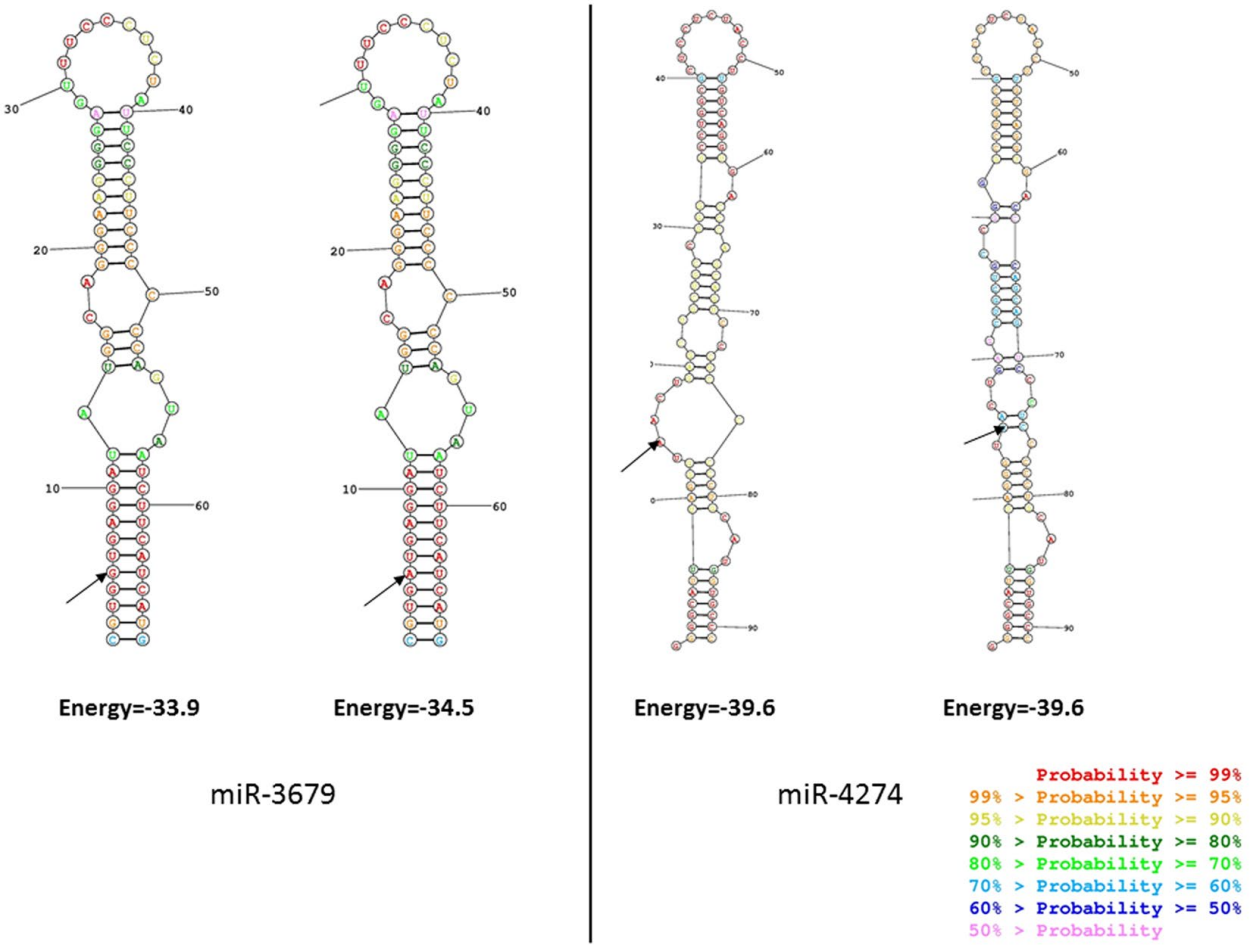
Predicted changes in secondary structure provoked by the BMD-associated SNPs according to RNAstructure web server. Black arrows tag the corresponding allele for rs6430498 in the miR-3679 and rs12512664 in the miR-4274.

MiR-3679 and miR-4274, which harbored BMD-associated SNPs in their pre-miRNA sequence (Fig. 2), were quantified by qPCR in order to compare the expression levels between OP and non-OP bone samples.

MiR-3679-5p was not detected in total bone samples or in cultured human osteoblast cells, but miR-3679-3p was present in both sample types. Therefore, only mature miR-3679-3p was assayed in the qPCR. Both miRNAs miR-3679-3p and miR-4274 were significantly overexpressed in the OP samples (Table 3).

**Table 3:** miRNA expression levels, comparison between osteoporotic and non-osteoporotic bone samples.

miRNA	Biological Group	RQ (Median)	IQR	*P* value
miR-3679	Osteoporotic	89.601	220.636	0.001
	Control	1.423	0.964	
miR-4274	Osteoporotic	144.268	318.409	0.001
	Control	1.197	2.154	

Abbreviations: RQ = Relative quantification; IQR = Interquartile Range.

Other bone miRNAs, miR-631 and miR-574-5p^1, 9^, were also tested in order to preclude a non-specific phenomenon related to a general upregulation of gene expression. No differences were found between osteoporotic and non-osteoporotic bone samples (p = 0.626 and p = 0.183 respectively).

### Association analysis of miRNA expression levels with genotypes of BMD-associated SNPs

**Figure 3:**
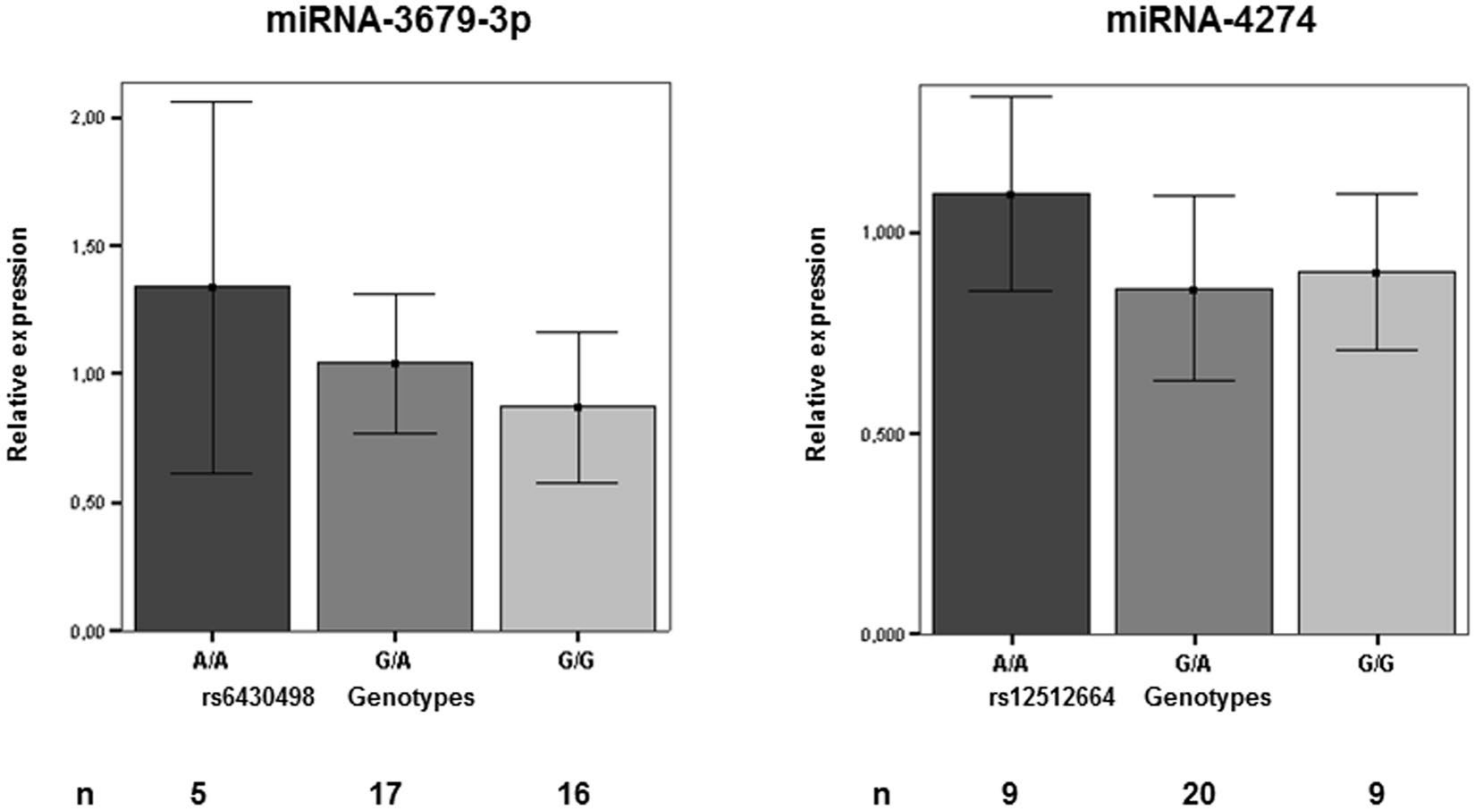
Correlation between miRNA expression levels and genotypes for miR-3679 and miR-4274. MiRNA expression levels are represented as a mean ± SD of the relative expression in Real-Time PCR. U6 was used for normalization. Samples of 38 human primary osteoblasts were used for experiments. (n) is the number of samples for each genotype group.

Human primary osteoblasts (n = 38) were cultured for DNA and RNA extraction and sorted by genotype for both rs6430498 and rs12512664. Association between expression levels of mature miRNA miR-3679-3p and miR-4274 with its corresponding own genotype was analyzed. A significant correlation was observed between miRNA levels and the genetic variant (Fig. 3). The A allele for both SNPs was associated with higher expression of each corresponding miRNA (miR-3674; log-additive model; *p* value = 0.015; recessive model; *p* value = 0.03, and miR-4274; recessive model; *p* value = 0.013).

Additionally, in order to corroborate that the differences among expression levels are due to genotypes “per se” and not for other cellular circumstances, other bone-related miRNAs^1, 9^ were checked in these cells. Expression levels of miR-320a, and miR-22-3p were measured and no differences were found irrespective of genotypes. Moreover, as a sensitivity test, we analyzed the miR-3679-3p and miR-4274 expression levels separated according to the exchanged genotype amongst both miRNAs (rs12512664 and rs6430498 respectively). Once again, no differences were found in both miRNA analyzed (miR-3679-3p; rs12512664: p-value = 0.713 and miR-4274; rs6430498: p-value = 0.645).

### Bioinformatic analysis

A subtle effect of the BMD-associated SNPs on secondary pre-miRNA structure was detected. The genetic variants rs6430498 and rs12512664 did not provoke evident changes in the loop formation on the RNAstructure web server (Fig. 2).

Among predicted targets for miR-3679-3p are *WHSC1/WHSC1L1, THRAP2, SMAD2, LRP6, SOX4, WNT7A, and MGAT5B*. The miR-3679 is encoded within MGAT5 gene. The protein encoded by this gene, the mannosyl (alpha-1,6-)-glycoprotein beta-1,6-N-acetyl-glucosaminyltransferase, is one of the most important enzymes involved in the regulation of the biosynthesis of glycoprotein oligosaccharides. The most significant pathways regulated by this miRNA is the glycerophospholipid metabolism (p-value = 0.02) and MAPK signaling pathway (p-value = 0.049).

Predicted targets for miR-4274 are *BTF3, LRRC1, SOCS5, RAB10*, *FGR, RABGEF2* and *ESRRG* genes. ErbB (p-value = 0.002), proteoglycan in cancer (p-value = 0.006) and mTOR (p-value = 0.01) signaling pathways are the top pathways involving gene targets for this miRNA.

### *In-vitro* assessment of osteoblast activity and matrix mineralization

hOBs were treated with miR-3679-3p and miR-4274 mimics or inhibitors and their respective controls for investigating their role in osteoblast function (Supplemental Fig. 1).

Inhibition of miR-3679-3p significantly enhanced 10% matrix mineralization levels at 28-day culture (Inhibitor: 1.64 ± 0.021; control: 1.49 ± 0.056; *p* value = 0.004). On the other hand, overexpression of miR-3679-3p in hOBs weakly reduces matrix mineralization although differences were not significant (Mimic: 1.53 ± 0.085; control: 1.68 ± 0.256; *p* value = 0.589). No significant differences in alkaline phosphatase (ALP) activity and cell proliferation were observed after miR-3679-3p transfection.

For miR-4274 transfections, no changes in any parameter evaluated were found.

## Discussion

Two osteoblast-related microRNAs, miR-3679 and miR-4274, were found to be associated with femoral neck BMD and overexpressed in OP fractured bone. These miRNAs harbor genetic variants in their pre-miRNA sequences that were correlated with the miRNA expression levels in human osteoblasts. Finally, inhibition of miR-3679-3p in human osteoblastic cells increased matrix mineralization. The lower density of genetic variants in miRNA genes compared to other non-coding genomic regions suggests that SNPs can have a remarkable biological role in pri-miRNAs by differential regulation of their target genes^10^. Hence, we chose genetic variants within the pri-miRNA sequences for their putative functionality. Moreover, the BMD-associated miR-SNPs in our study were located in the pre-miRNA molecules, which have been described to exhibit strong selective constraints^11, 12^.

SNPs within the pri-miRNA sequence can affect the miRNA maturation in multiple aspects, modifying the hairpin structure or changing the binding affinity of biogenesis enzymes to the miRNA hairpin. Moreover, variants can affect alternative cleavage sites of biogenesis enzymes, producing novel isomiRs or changing their frequency. These changes in maturation can result in altered expression of the functional miRNA, resulting in deregulation of target genes. Hence, several studies have suggested that functional SNPs in pri-miRNAs affect the processing and expression levels of mature miRNAs^13^.

Two mir-SNPs, rs6430498 and rs12512664, within the pre-miRNA sequence of miR-3679 and miR-4234, respectively, were associated with FN BMD in the OSTEOMED2 cohort. Bioinformatic analysis of the hairpin structure of these BMD-associated miRNAs showed no striking effect on its secondary structure caused by allelic changes but we cannot rule out that the expression was altered by affecting the affinity binding of the processing enzymes or their accessory proteins. In this regard, other studies have demonstrated that miR-SNPs in pre-miRNA sequences lead to altered levels of mature miRNA *in vivo* even though changes in its secondary structure are not predicted by RNAfold program^14^.

In our study the AA genotype for both rs6430498 and rs12512664 showed higher expression levels of miR-3679-3p and miR-4274 in hOBs, respectively. Moreover, these miRNAs were overexpressed in OP bone samples. Consistently with miR-3679-3p overexpression in OP fractured bone, transfection results suggest that miR-3679-3p negatively regulates matrix mineralization by osteoblasts which could lead to an OP phenotype. Further supporting these results, the A allele for both SNPs was associated with lower FN BMD levels.

In overall, predicted targets and pathways involving miR-3679-3p belong to cell cycle, differentiation and tissue development, suggesting a dysfunction in bone remodeling in the osteoporotic bone. In agreement with these data, an impaired mineralization was observed when miR-3679-3p was overexpressed in osteoblasts. In contrast, analysis of targeted genes and pathways for miR-4274 suggests that this miRNA could be involved, broadly, in vesicular and membrane trafficking, cell migration and adhesion, and cell metabolism. That would explain why changes in the assayed parameters were not detected after miR-4274 transfection in osteoblasts.

Altogether, these findings suggest that these variants can play a role in bone metabolism. Since information about these two miRNAs is very scarce, this study could be considered as a preliminary approach presenting miR-3679-3p and miR-4274 within the complex network of bone regulation.

Our study is strengthened by the extremely careful in control of potentially confounding characteristics among bone samples in terms of age, sex, BMI and metabolic diseases highly prevalent in elderly population^15, 16^. Moreover, we excluded from the study samples from patients treated with hormones and other anti-osteoporotic drugs that could alter the miRNA expression in bone cells^17^. These strict inclusion criteria support that miRNAs detected in our study can be more reliably considered involved in the osteoporotic phenotype.

One limitation is the use of osteoarthritic samples as control group. Due to obvious ethical reasons the collection of bone from healthy individuals is not allowed. However, in an attempt to minimize this potential limitation, we obtained the bone samples from a location distant from the interface between bone and cartilage and, therefore, as far away as possible from the osteoarthritic lesion. Moreover, although both miRNAs have found overexpressed in OP bone tissue, it cannot be attributed to the SNP regulation in these samples since this could not be analyzed due to lack of bone sample’s availability. Other limitation of the study is the need to replicate the association results in other cohorts because these miR-SNPs lacked linkage information with other tag-SNPs related to bone phenotypes. However, this is the first time that two genetic variants in human osteoblast-related miRNAs have been associated with FN BMD in an extensive cohort of postmenopausal women, and this association was functionally demonstrated in bone samples.

In conclusion, two putative functional SNPs in the pre-miRNA sequence of miR-3679 and miR-4274 were associated with femoral neck BMD. In both cases, the allele associated with lower BMD was correlated with higher expression levels of mature miRNA in human osteoblastic cells. Moreover, both miRNAs were overexpressed in OP fractured bone. Our results open new exploratory avenues for future studies in the bone field.

## Methods

### Ethics Statement

The study protocols for obtaining DNA from blood samples, bone tissue samples and primary osteoblasts were approved by the CEIC-Parc de Salut MAR, the coordinating center (Registry number 2010/3882/I). Methods were carried out in accordance with the approved guidelines by the CEIC-Parc de Salut MAR. The approved protocols were explained to potential study participants, who provided written informed consent before being included in the study. All studies were carried out in accordance with the principles of the Declaration of Helsinki as revised in 2008.

### Study subjects

Genetic association analysis was performed in an observational, clinical cohort study (OSTEOMED2) of patients recruited from 14 medical centers in several regions of Spain. All patients were consecutive, unselected, postmenopausal women attended in an outpatient clinic. Patients were prospectively recruited regardless of BMD value (Table 4). Exclusion criteria were any history of metabolic or endocrine disease, chronic renal failure, chronic liver disease, malignancy (except superficial skin cancer), Paget’s disease of bone, malabsorption syndrome, and any anti-OP or bone-affecting treatment. In addition, women with early menopause (before the age of 40) were excluded from analysis.

**Table 4:** Baseline characteristics of the OSTEOMED2 cohort.

Patient characteristic	Mean ± SD	
	LS BMD n = 2183	FN BMD n = 2015
Age (years)	57.61 ± 9.26	58.80 ± 8.99
Age of menopause (years)	48.7 ± 3.94	48.7 ± 3.92
BMI (kg/m^2^)	26.56 ± 4.18	26.48 ± 4.13
BMD (g/cm^2^)	0.870 ± 0.16	0.707 ± 0.14

Abbreviations: BMI = body mass index; BMD = bone mineral density; LS = lumbar spine; FN = femoral neck.

BMD (g/cm^2^) was measured at the lumbar spine (LS) L2-L4 and at the non-dominant femoral neck (FN) using the dual-energy X-ray densitometers available in each participating center (Supplemental Table 1).

Whole bone samples for qPCR miRNA quantification were obtained from the transcervical region of the femoral neck of postmenopausal women undergoing hip replacement due to either OP fracture (n = 10) or osteoarthritis in the absence of osteoporosis (n = 10) (T-score measurements [mean ± SD]: 0.616 ± 0.523) (Table 2). Bone was taken from a location distant from the interface between bone and cartilage and, therefore, as far away as possible from the osteoarthritic lesion. Again, none of the participants had a history of metabolic or endocrine disease, chronic renal failure, chronic liver disease, malignancy, Paget’s disease of bone, malabsorption syndrome, hormone replacement therapy, anti-resorptive or anabolic agents, oral corticosteroids, anti-epileptic drugs, or treatment with lithium, heparin, or warfarin.

### SNPs selection in pri-miRNA sequences

In the first step, 9 candidate genes were selected for their well-known function in bone regulation: *ESR1*, *TGFB2*, *PTH1R*, *RUNX2*, *IL6R*, *LRP5*, *IL6*, *VDR*, and *CYP24A1* ^18–23^ (Table 1). Then, miRNAs that bind to the mRNA 3′UTR of these genes were identified using the microRNA.org website^24^ (http://www.microrna.org), TargetScan Human (http://www.targetscan.org) and PicTar (http://pictar.mdc-berlin.de). The algorithm used is based on sequence complementarity, binding energy of the miRNA–target duplex, evolutionary conservation of the target site sequence and target position in aligned UTRs of homologous genes. Some listed miRNAs target more than one bone-related gene, as for example, miR-130b, miR106b, miR148b and miR-93 that target both *ESR1* and *TGFB2* genes. These miRNAs were located in *ESR1* target miRNAs in order to simplify the Table 1. Next, miRNAs with the highest expression levels in primary human osteoblasts (hOB) were selected (Table 1) according to results obtained in a previous miRNA array performed in our group (GEGSE74211; http://www.ncbi.nlm.nih.gov/geo/query/acc.cgi? acc = GSE74211)^1^. Finally, SNPs in the pri-miRNA sequences were searched using the Ensembl (www.ensembl.org), UCSC Genome Browser on Human Dec. 2013 (GRCh38/hg38) Assembly^25^ (http://genome.ucsc.edu/), and HapMap (www.hapmap.org) databases.

Only those SNPs with a minor allele frequency (MAF) in Utah residents with ancestry from northern and western Europe (CEU) > 0.01 were included in the study; SNPs lacking published MAF were validated in participants from the BARCOS cohort^8^ (included in OSTEOMED2) by means of PCR-RFLP, genotyping or Sanger sequencing.

### Polymorphism genotyping

DNA was obtained from peripheral blood collected in EDTA tubes and genotyping was performed in LGC Genomics platform using KASPar v4.0 genotyping systems. To ensure genotyping quality, a random sample (5% of the total number of samples) was also genotyped in a separate control plate. There was 100% concordance between these results.

### Human osteoblasts culture

Human primary osteoblasts (hOB) were obtained from trabecular bone of patients who underwent knee or hip replacement due to osteoarthritis. Bony tissue was cut up into small pieces, washed in phosphate buffered solution (PBS, Gibco by Life Technologies; Paisley, UK) to remove non-adherent cells, and placed on a 140 mm culture plate. Samples were incubated in hOB medium: Dulbecco’s Modified Eagle Medium (DMEM; Gibco; Invitrogen, Paisley, UK), supplemented with 10% fetal calf serum (FBS; Sigma-Aldrich; St. Louis, USA), 100 U/ml penicillin/streptomycin (Sigma-Aldrich; St. Louis, USA), 0.4% fungizone (Gibco by Life Technologies; Paisley, UK), and 100 ug/ml ascorbic acid (Sigma; Steinheim, Germany). Alkaline Phosphatase (ALP) activity and osteocalcin gene expression were measured in order to confirm the osteoblastic phenotype (data not shown). All experiments were performed at maximum passage 2.

Thirty eight samples were grown in parallel: one for DNA extraction and the other for RNA extraction. Additionally, 11 hOB samples were used for Alizarin Red, ALP and MTS assays.

### Human osteoblast DNA extraction and sequencing

DNA was extracted from cultured hOBs using the Wizard® Genomic DNA Purification Kit (Promega) according to manufacturer's instructions. Genotypes for rs6430498 and rs12512664 were assessed by Sanger sequencing using the BigDye® Terminator v3.1 (Applied Biosystems) in the genomic services of Universitat Pompeu Fabra. Primers (Sigma-Aldrich) were designed using the Primer3 imput (v. 0.4.0) and the UCSC website: for rs6430498 in miR-3679, F: 5′-CGGTGAGGAGTTTTCTGAATG-3′ and R: 5′-CACCAAGCATAATAGCTAAAAATCAA-3′ (fragment size: 400 bp) and for rs12512664 in miR-4274, F: 5′-CATCCACTTTGGGGAGAAGT-3′ and R: 5′-CCAAGGTACCACTGCCTCAT-3′ (fragment size: 392 bp).

### RNA extraction of osteoblasts and total bone

miRNA-enriched fraction from hOB cultures was extracted using MiRNeasy mini kit and RNeasy MinElute Cleanup (Qiagen) according to manufacturer instructions.

For RNA extraction of total bone, a piece of tissue was cut out and added to QIAzol Lysis Reagent (Qiagen), then homogenized for 5 min using the TissueLyser system. Chloroform was added to each sample, followed by centrifugation for 15 min (12000 g). The upper water phase was collected and the extraction continued according to manufacturer’s instructions. The quality of the total RNA was verified by an Agilent 2100 Bioanalyzer profile. The concentration of the purified RNA was analyzed on a spectrophotometer (Nanodrop, Thermo Fisher Scientific Inc).

### miRNA quantification by qPCR

Using the miScript II RT kit (Qiagen), 1 µg of total RNA was reverse-transcribed in 20 μl reactions. cDNA from total bone was diluted x8 and cDNA from hOBs was diluted x16; in both cases, 2 µl were assayed in 10 µl PCR reactions in 384-well plates using MiScript Syber Green PCR kit according to the protocol. The sequence of the mature miRNAs selected, according to the mirBase web site, was used as a forward primer and the Universal primer as a reverse. U6 snRNA was used as the reference gene for normalization. Amplification was performed in a QuantStudio 12 K Flex Real-Time PCR (Applied Biosystems), and the Expression Suite software was used both for determination of relative quantification (RQ) (by 2^−ΔΔCt^ method) and for melting curve analysis.

### Bioinformatics analyses of BMD-associated miRNAs

Gene target prediction was assessed using the following six programs: PicTar (http://pictar.mdc-berlin.de), TargetScan Human (http://www.targetscan.org), miRDB (http://mirdb.org), MiRanda (http://www.microrna.org), DIANA-TarBase (http://diana.imis.athena-innovation.gr), and miRTarBase (http://mirtarbase.mbc.nctu.edu.tw). The DIANA-mirPath web-based computational tool^26^ was used to identify molecular pathways potentially altered by the intersection of miRNAs differentially expressed in fractured bone.

miRNASNP database^10^, RNAstructure (http://rna.urmc.rochester.edu/RNAstructureWeb) and RNAfold software (http://rna.tbi.univie.ac.at/cgi-bin/RNAfold.cgi) were used to predict the effect of the variants on the miRNA secondary structure. These predictions are based on the assumption that variants can destabilize the hairpin and therefore reduce the mature miRNA levels.

### Cell transfection

Primary hOB cells were seeded at the following conditions: 96-well plate at 12.000 cells/well for MTS and ALP activity assays and 24-well plate at 45.000 cells/well for Alizarin Red quantification.

Once cells reached 60–70% of confluence, transient transfections were performed using *mir*Vana mimics and inhibitors of both hsa-miR-3679-3p and hsa-miR-4274. *Mir*Vana™ miRNA Mimic Negative Control #1 and *mir*Vana™ miRNA Inhibitor Negative Control #1 were used as controls. All products were purchased from Ambion® Life Technologies. Mimics and controls mimics were used at 100 nM and inhibitors and control inhibitors at 400 nM. In order to monitor transfection efficiency, miRIDIAN microRNA Mimic Transfection Control with Dy547 (Dharmacon) was transfected into the cells at the same conditions as the miRNAs tested. Transfection of miRNAs was carried out using Lipofectamine RNAiMAX (Invitrogen; Carlsbad, USA) according to the manufacturer’s instruction.

### Alizarin Red quantification

To induce osteoblastic mineralization, hOBs (n = 4) were cultured during 28 days with hOB medium supplemented with 5 mM β-glicerophosphate (Sigma-Aldrich, St Louis, MO, USA). Cells were transfected with both mimics and inhibitors of miR-3679-3p and miR-4274, and their corresponding controls at day 1, and day 14 after seeding. During the cell culture time period, the medium was changed once every 3 days.

At day 28, cells were stained with alizarin red to quantify mineralized nodules. At this time point, media was removed from the cell monolayer and gently washed 3 times with PBS. The cells were then fixed in 10% buffered formalin for 10 minutes at room temperature. Fixative was removed and cultures were washed in PBS. The cell layer was stained with 2% Alizarin-S (Sigma-Aldrich, St Louis, MO, USA) at ~pH 4.2 for 20 minutes. Cell preparations were washed with PBS to eliminate nonspecific staining. To quantify calcium deposition, the dye was leached by the addition of 10% cetylpyridinium chloride until all of dye had been drawn. Optical density was then quantified by spectrophotometry at 550 nm, using 10% cetylpyridinium chloride as a blank reference.

### ALP activity assay

ALP activity was measured 48 hours after miRNA transfection of hOBs (n = 4) using the Alkaline Phosphatase Assay Kit (Colorimetric) (Abcam; Cambridge, UK) according to the manufacturer’s instructions.

### Cell viability assay (MTS)

Viable cells were determined 48 hours post-transfection of hOBs (n = 3) using the CellTiter 96® AQueous One Solution Cell Proliferation Assay (Promega; WI, USA), according to the manufacturer’s instructions.

### Statistical methods

Hardy-Weinberg equilibrium (HWE) was calculated using the chi-square test. Multiple linear regression models were fitted to assess the association between genotyped SNPs and BMD. Log-additive, dominant and recessive models were tested for each SNP association analysis. Potential confounders considered for adjustment were densitometer devices, body mass index (BMI) and age. Association analyses were performed using R software version 2.13.2 with the *SNPassoc*, *foreign*, *gdata* and *multtest* packages.

Mann-Whitney U test was performed for OP and non-OP group comparisons in the SPSS v.12.0 for Windows. This test was also used for treatment comparisons between miRNA mimics or inhibitors and their respective controls.

Linear regression was used to analyze the association between miRNA expression levels and sample genotypes. Log-additive, dominant and recessive models were tested for each miRNA analysis. Correlation analyses were performed using R software version 2.13.2 with the *SNPassoc* and *foreign* packages.

All analyses were two-tailed, and p-values < 0.05 were considered significant.

## Electronic supplementary material

Supplemental Figure 1
