# Supplementary material for: SNPs in bone-related miRNAs are associated with the osteoporotic phenotype
Source: Sci Rep. 2017 Mar 31;7:516. doi: 10.1038/s41598-017-00641-7 (PMC5428869; doi:10.1038/s41598-017-00641-7)
Supplement: Supplementary file 1 — Supplemental Figure 1 [file 41598_2017_641_MOESM1_ESM.pdf]

SNPs in bone-related miRNAs are associated with the osteoporotic phenotype

Laura De-Ugarte[1], Enrique Caro-Molina[1], Maria Rodríguez-Sanz[1], Miguel Ángel García-Pérez [2], José M. Olmos[3], Manuel Sosa-Henríquez[4], Ramón Pérez-Cano[5], Carlos Gómez-Alonso[6], Luis Del Rio[7], Jesús Mateo-Agudo[8], José Antonio Blázquez-Cabrera[9], Jesús González-Macías[10], Javier del Pino-Montes[11], Manuel Muñoz-Torres[12], Manuel Diaz-Curiel[13], Jorge Malouf[14], Antonio Cano[15], José Luis Pérez-Castrillon[16], Xavier Nogues[1], Natalia Garcia-Giralt[1*], Adolfo Diez-Perez[1].

[1]IMIM (Hospital del Mar Medical Research Institute), Universitat Autònoma de Barcelona, RETICEF (ISCII), Barcelona

[2]Department of Genetics and Institute of Health Research INCLIVA, University of Valencia, Valencia

[3]Department of Internal Medicine, Hospital Universitario Marqués de Valdecilla-IDIVAL/Hospital de Torrelavega, Universidad de Cantabria. RETICEF, Santander

[4]Unidad Metabólica Ósea, Hospital Universitario Insular, Universidad de Las Palmas de Gran Canaria.

[5]Departamento de Medicina (USE), UGC Medicina Interna, Hospital Universitario Virgen Macarena, Sevilla

[6]Servicio de Metabolismo Óseo y Mineral, Hospital Universitario Central de Asturias, Oviedo

[7]CETIR Grup Mèdic ,RETICEF, Barcelona.

[8]Servicio COT, Hospital Universitario Miguel Servet, Zaragoza

[9]Servicio de Medicina Interna, Complejo Hospitalario Universitario de Albacete, Albacete

[10]Departamento de Medicina Interna, H. Marqués de Valdecilla, Universidad de Cantabria, IDIVAL, RETICEF, Santander

[11]Servicio de Reumatología. Hospital Universitario de Salamanca. RETICEF (ISCIII). IBSAL (Biomedical Research Institute of Salamanca), Salamanca

[12]UGC Endocrinologia y Nutrición. Hospital Universitario San Cecilio. Granada, RETICEF, Ibs Granada

[13]Unidad de Enfermedades Metabólicas Óseas. Servicio de Medicina Interna. Fundacion Jimenez Diaz, Madrid

[14]Hospital de la Santa Creu I Sant Pau. Institut d'Investigació Biomèdica Sant Pau, Barcelona.

[15]Department of Pediatrics, Obstetrics and Gynecology, University of Valencia, INCLIVA, Valencia

[16]Hospital Universitario Río Hortega, Valladolid

Corresponding author: Natalia Garcia-Giralt

e-mail: ngarcia@imim.es

Phone: 0034 933160497

Fax: 0034 933160410

Supplemental Table 1. Participant centers of the OSTEOMED2 cohort and DXA devices

Medical center	Densitometer
C. Hospitalario Universitario de Albacete	Norland System XR-26 (Norland Medical Systems Inc.; Fort Atkinson, WI, USA)
CETIR Centre Médic	GE-Lunar, iDXA (General Electric Healthcare, Iinc)
Complejo Hospitalario Universitario de Granada	QDR 4500 (Hologic,Waltham, MA, USA)
Fundacion Jimenez Diaz	QDR 4500 C (Hologic,Waltham, MA, USA)
Hospital de Sant Pau	QDR Discovery (Hologic,Waltham, MA, USA)
Hospital del Mar	QDR 4500 SLR (Hologic,Waltham, MA, USA)
Hospital Universitario Central de Asturias	Hologic Discovery A (Hologic,Waltham, MA, USA)
Hospital Universitario de Salamanca	QDR 4500 SLR (Hologic,Waltham, MA, USA)
Hospital Universitario Insular	QDR 4500 SLR (Hologic,Waltham, MA, USA)
Hospital Universitario Marqués de Valdecilla	QDR 4500 W (Hologic,Waltham, MA, USA)
Hospital Universitario Miguel Servet	LUNAR DPX-NT densitometer (General Electric Company, Madison, MI, USA)
Hospital Universitario Virgen Macarena	Hologic "Discovery" (Hologic,Waltham, MA, USA)
Hospital Universitario Río Hortega	Lunar Prodigity Primo (General Electrics Helthcare, USA)
Institute of Health Research INCLIVA	Norland XR-36 (Norland Medical Systems Inc.; Fort Atkinson, WI, USA)<br>Lunar DPX (GE Lunar Corporation, Madison, WI, USA)

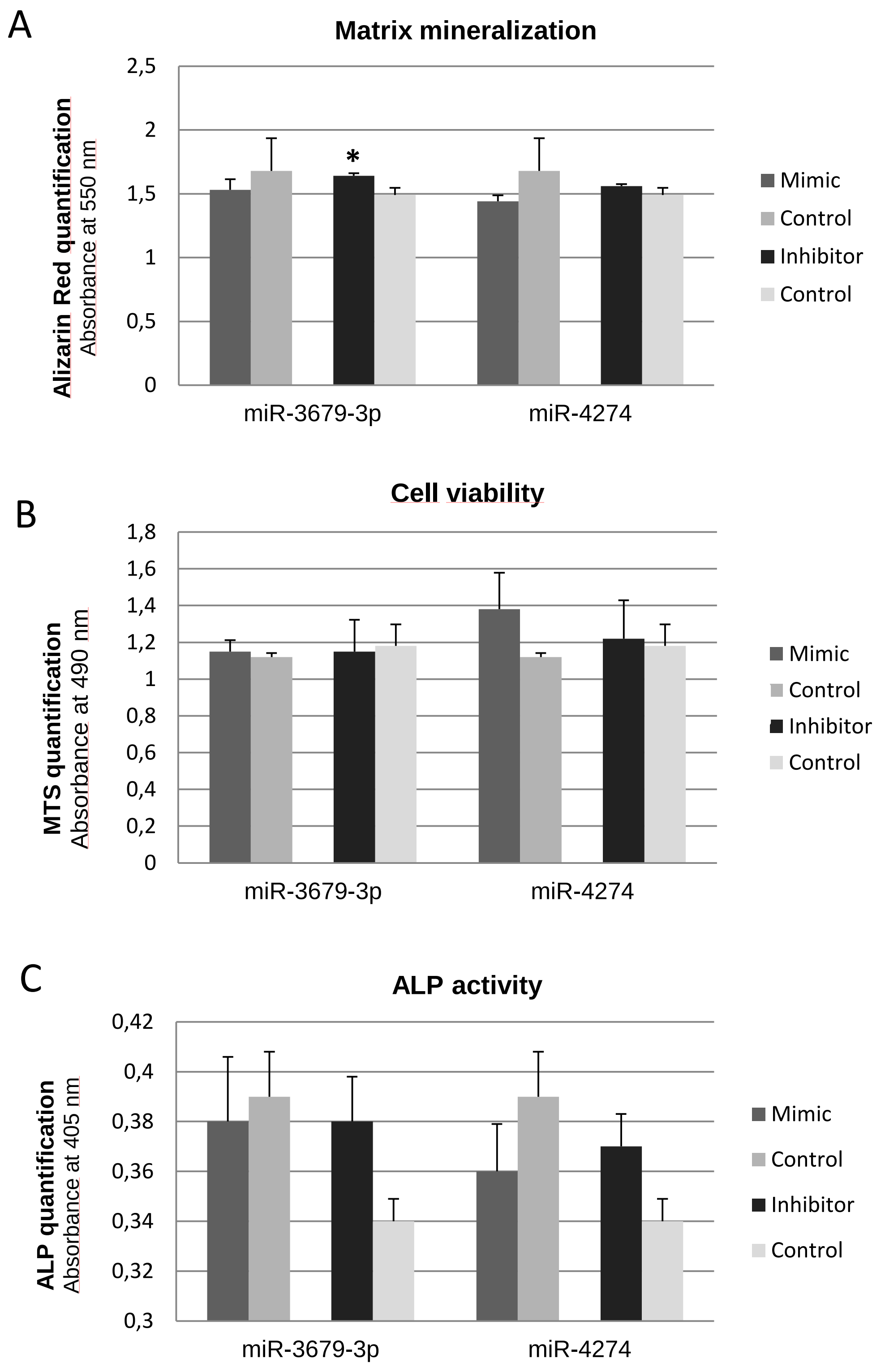

Supplemental Fig. 1. In-vitro assessment of osteoblast activity and matrix mineralization after hOB transfection with mimics or inhibitors of miR-3679-3p and miR-4274: A) Matrix mineralization evaluated by using alizarin red staining and quantification. B) Cell viability evaluated by using MTS assay. C) ALP activity evaluated by using Alkaline Phosphatase Assay. ***** $p<0.05$
